# Platelets in ITP: Victims in Charge of Their Own Fate?

**DOI:** 10.3390/cells10113235

**Published:** 2021-11-19

**Authors:** Vivianne S. Nelson, Anne-Tess C. Jolink, Sufia N. Amini, Jaap Jan Zwaginga, Tanja Netelenbos, John W. Semple, Leendert Porcelijn, Masja de Haas, Martin R. Schipperus, Rick Kapur

**Affiliations:** 1 Department of Hematology, Haga Teaching Hospital, 2545 AA The Hague, The Netherlands; v.nelson@hagaziekenhuis.nl (V.S.N.); S.Amini@hagaziekenhuis.nl (S.N.A.); t.netelenbos@hagaziekenhuis.nl (T.N.); 2 Sanquin Research, Department of Experimental Immunohematology, Landsteiner Laboratory, Amsterdam UMC, University of Amsterdam, 1066 CX Amsterdam, The Netherlands; a.jolink@sanquin.nl (A.-T.C.J.); m.dehaas@sanquin.nl (M.d.H.); 3 Department of Hematology, Leiden University Medical Center (LUMC), 2333 ZA Leiden, The Netherlands; j.j.zwaginga@lumc.nl; 4 CCTR, Sanquin Blood Supply, 1066 CX Amsterdam, The Netherlands; 5 Division of Hematology and Transfusion Medicine, Lund University, 221 84 Lund, Sweden; john_w.semple@med.lu.se; 6 Clinical Immunology and Transfusion Medicine, Office of Medical Services, 221 84 Lund, Sweden; 7 Sanquin Diagnostic Services, Department of Immunohematology Diagnostics, 1066 CX Amsterdam, The Netherlands; l.porcelijn@sanquin.nl; 8 Department of Hematology, University Medical Center Groningen (UMCG), 9713 GZ Groningen, The Netherlands; m.r.schipperus@umcg.nl

**Keywords:** ITP, platelet immune functions, platelet microparticles

## Abstract

Immune thrombocytopenia (ITP) is an autoimmune bleeding disorder. The pathophysiological mechanisms leading to low platelet levels in ITP have not been resolved, but at least involve autoantibody-dependent and/or cytotoxic T cell mediated platelet clearance and impaired megakaryopoiesis. In addition, T cell imbalances involving T regulatory cells (Tregs) also appear to play an important role. Intriguingly, over the past years it has become evident that platelets not only mediate hemostasis, but are able to modulate inflammatory and immunological processes upon activation. Platelets, therefore, might play an immuno-modulatory role in the pathogenesis and pathophysiology of ITP. In this respect, we propose several possible pathways in which platelets themselves may participate in the immune response in ITP. First, we will elaborate on how platelets might directly promote inflammation or stimulate immune responses in ITP. Second, we will discuss two ways in which platelet microparticles (PMPs) might contribute to the disrupted immune balance and impaired thrombopoiesis by megakaryocytes in ITP. Importantly, from these insights, new starting points for further research and for the design of potential future therapies for ITP can be envisioned.

## 1. Introduction

Immune thrombocytopenia (ITP) is an acquired autoimmune disease characterized by low platelet counts (<100 × 10^9^/L) due to increased platelet destruction and impaired platelet production [1,2]. Patients with ITP are at risk of clinically significant bleedings. Their bleeding tendency ranges from petechiae and purpura to, in rare cases, intracranial hemorrhages [2,3]. In addition, patients with ITP report a reduced health-related quality of life as they suffer from fatigue and concerns about their condition [4]. The course of ITP differs between children and adults, as pediatric patients are more likely to experience spontaneous remissions [5]. Hence, the first-line management of pediatric ITP often consists of a period of careful observation rather than corticosteroids [5]. Adult patients, however, have a higher likelihood of progression to chronic ITP and often require multiple lines of therapy [5]. Therapies for ITP include corticosteroids, intravenous immunoglobulin (IVIG), rituximab, thrombopoietin receptor agonists (TPO-RAs) and splenectomy [5]. Moreover, combinations of therapies are considered in patients with refractory ITP [5]. The clinical presentation of ITP and the responsiveness of patients with ITP to different treatments is very heterogeneous [2]. Similarly, the pathophysiological mechanisms involved in ITP are thought to be diverse, complex and are not yet fully elucidated [2,3]. Central to ITP pathogenesis is the loss of self-tolerance, leading to both autoantibody formation and abnormal CD8+ T cell responses [1,2,3,6]. These platelet-autoantibodies can induce Fc-dependent platelet destruction via phagocytosis, complement activation and apoptosis [1,2,3]. Activation of the classical complement pathway, for example, has been described in approximately 60% of the patients with ITP [7]. Nonetheless, 20% of patients with ITP have no clinically detectable autoantibodies, indicating that, at least for a subgroup of patients, autoantibody-independent pathways such as platelet destruction by CD8+ cytotoxic T cells are involved [1,2,3,6,8,9]. Furthermore, Fc-independent pathways, such as the clearance of desialylated platelets through the hepatic Ashwell-Morell receptor, have also been described to contribute to the enhanced platelet clearance in ITP [3,10]. In addition to increased platelet destruction, the production of platelets is decreased in ITP due to autoimmune-related impaired thrombopoiesis by megakaryocytes in the bone marrow associated with insufficient upregulation of endogenous thrombopoietin (TPO) levels [1,3,11].

**Figure 1:**
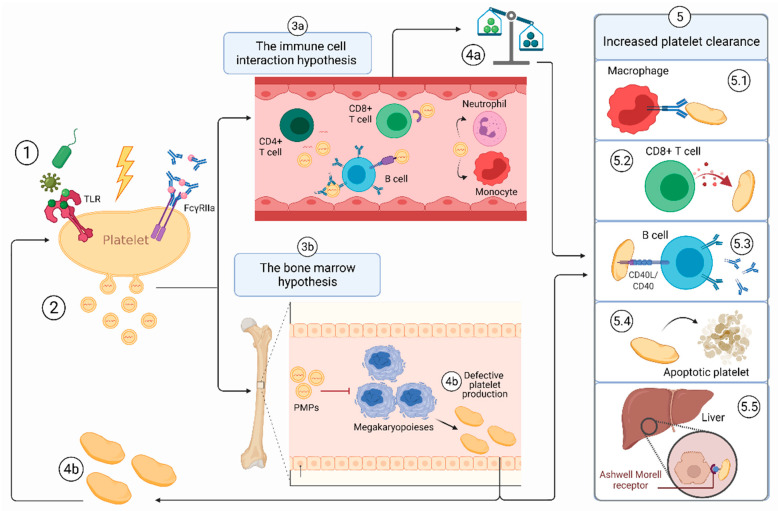
Two potential pathways in which PMPs may participate in the pathogenesis of ITP. (**1**) A platelet initially gets hit by a trigger, e.g., pathogens activating platelet Toll-like receptors (TLRs) or binding of immune complexes/ autoantibodies to the platelet FcγRIIa or other forms of (shear) stress. (**2**) The first hit causes the platelet to release higher numbers of PMPs and PMPs with aberrant cargo (ITP-PMPs). (**3a**) The immune cell interaction hypothesis: PMPs may interact with various immune cells including B cells, T cells, neutrophils and dendritic cells (DCs). (**4a**) These immune cell interactions may contribute to the disrupted T cell-immune balance and the loss of immune tolerance in ITP. (**3b**) The bone marrow hypothesis: PMPs may infiltrate the bone marrow and impair the function of megakaryocytes leading to disrupted megakaryopoiesis. (**4b**) Less (and dysfunctional) platelets are produced by abnormal megakaryocytes (see Figure 2). Again, these dysfunctional platelets shed ITP-PMPs. (**5**) The changes in the immune balance (**4a**) and megakaryopoiesis (**4b**) may promote increased platelet clearance through several mechanisms (**5.1**–**5.5** *). Platelets with dysfunctional properties are produced by abnormal megakaryocytes possibly causing them to be more easily cleared from the circulation. Besides the properties listed in Figure 2, these dysfunctional platelets might also have more phosphorylcholine exposure and stimulate cytotoxic CD8+ T cell responses, leading to **5.1** and **5.2**, respectively. * The mechanisms involved in the increased platelet clearance in ITP include (**5.1**) increased autoantibody mediated phagocytosis, (**5.2**) increased CD8+ cytotoxic T cell destruction, (**5.3**) aberrant CD40L-CD40 interaction with B cells leading to more platelet-antibody production, (**5.4**) increased platelet apoptosis and (**5.5**) increased clearance of desialylated platelets by the Ashwell-Morell receptor. Created with BioRender.com.

Within the past decade, it has become increasingly clear that platelets are not only important for hemostasis, but also exhibit several non-hemostatic immunological functions [12,13,14,15]. Platelets are able to capture pathogens and interfere in viral or bacterial infections [12,13,14,15]. Furthermore, platelets can interact with other immune cells and are able to modulate immune responses in various ways such as B cell activation through expression of, for example, (s)CD40L molecules [12,13]. Moreover, platelets can orchestrate inflammatory and cellular responses by releasing platelet microparticles (PMPs) in the circulation [12,13,15]. Recent evidence has also outlined the importance of the immunological functions of platelets in the etiology and pathophysiology of other autoimmune diseases including systemic lupus erythematosus (SLE) and rheumatoid arthritis (RA) [16,17]. Thus far, platelets have been mainly acknowledged for their hemostatic role in ITP and relatively little has been described about their potential immunological role(s). Hence, in this review we will discuss how platelets themselves might be able to elicit or aggravate the disrupted immune response underlying their own destruction in ITP. First, we will highlight how platelets may affect inflammation and interact with several immune cells in ITP. Second, we propose two new pathways in which PMPs contribute to the disrupted immune balance and impaired thrombopoiesis by megakaryocytes in ITP (Figure 1). Finally, we aim to provide new starting points for further research regarding the immune modulations by platelets in ITP, which may lay the basis for the development of potential new diagnostic and therapeutic strategies in ITP. 

## 2. Hypothesis 1: Platelets and Their Immune Functions in ITP

### 2.1. Platelet Activation and Immune Functions

Growing evidence has outlined the importance of platelets as both immune sensing and immune effector cells in innate and adaptive immunity. For a more detailed reading on these functions in general, we refer to recent reviews on this topic [12,14,15,18]. Platelet activation is considered to be crucial for platelets to carry out their immunological functions [13,14]. In ITP, a significant proportion of the autoantibodies target platelet glycoprotein (GP) receptors that are responsible for platelet activation, such as GPIIb/IIIa and GPIb/IX/V complexes. So far, however, conflicting results have been published about platelet activation in ITP. Platelet activation has been reported to be either increased, within normal range or decreased in ITP [19,20,21]. Platelet activation, rather than platelet counts, has been suggested to be a possible predictor of bleeding risk [21,22,23]. Although the nature and degree of this association is not yet fully elucidated, these studies imply that the function of platelets may be altered in ITP. Psaila et al., for example, showed higher in vivo platelet reactivity in patients with ITP compared with equally thrombocytopenic patients with acute myeloid leukemia or myelodysplasia [22]. In addition, the platelet reactivity was lower in patients with ITP with bleeding compared with those without bleeding. Therefore, the autoantibodies in ITP may not only destroy platelets, but may also affect the function of the platelets remaining in the circulation [23,24]. Moreover, the impaired megakaryocyte-thrombopoiesis in ITP might not only lead to a quantitative impairment of platelet production, but also to the production of platelets with altered functions [20]. In addition, enhanced platelet clearance will increase the proportion of young and potentially different functioning platelets in the circulation [22]. Hence, the autoimmune-induced changes in the primary response capacities of platelets might influence platelet activation-dependent functions. In this regard, we suggest that the immunological functions of ITP platelets are likely also changed and might have a direct modulating influence on the pathogenesis and pathophysiology of ITP. In the following paragraphs, we will highlight and focus on the potential role of platelets in the initiation, progression and maintenance of inflammation and auto-immunity in ITP.

### 2.2. Platelets and Inflammation in ITP

Platelets can interact with viruses or bacteria in various ways. Firstly, platelets express Toll-Like Receptors (TLR2, 3, 4, 7 and 9), a family of pattern-recognition receptors which are able to recognize both pathogen- and damage-associated molecular patterns (PAMPs and DAMPs, respectively) [12,14,25]. Upon activation, platelet-TLRs initiate and orchestrate inflammatory responses. Platelet TLR-4, for example, has been shown to induce the formation of Neutrophil Extracellular Traps (NETs) in sepsis [26]. NETs are web-like chromatin complexes that are released by neutrophils in the circulation upon activation [27]. NETs are able to trap pathogens and elicit antimicrobial and inflammatory properties [19,27,28]. Aberrant NET formation (NETosis) has been described to propagate inflammation in various autoimmune diseases such as RA, SLE and anti-neutrophil cytoplasmic antibodies (ANCA)-associated vasculitis [19,28]. It has been speculated, for example, that NET formation can stimulate dendritic cells and autoreactive B cells in a TLR-9 dependent manner in patients with small vessel vasculitis [28]. In addition, platelet-driven NETosis has been described to occur in transfusion-related acute lung injury (TRALI) [29]. Two small cohort studies (15 patients with chronic ITP vs. 15 healthy controls, seven patients with acute ITP and 56 patients with persistent/chronic ITP vs. 30 healthy controls) have reported elevated markers of NETosis in ITP [19,30]. We hypothesize that NETosis, driven by platelets, could have a pro-inflammatory role in ITP. However, the available evidence about the presence of NETs or their contribution to the disrupted immune balance in ITP is not sufficient to currently conclude on such an association [19,30]. Moreover, the concept of platelet-driven NETosis in ITP raises several unanswered questions such as if NETosis may still be elevated in chronic severe thrombocytopenic patients and how NETosis may be stimulated in these patients. Several other ligands and functions of platelet-TLRs have been described recently such as activation of the NF-kappa-B pathway which can lead to changes in the secretory profile of platelets that could potentially influence inflammatory or immunological processes in ITP [12]. Further studies are needed to evaluate if and how platelet-TLRs are involved in ITP. Moreover, platelets are able to inhibit bacterial growth by encapsulating bacteria and releasing NET-inducing antimicrobial peptides [31]. ITP is closely related to inflammation as infections, both viral and bacterial, can trigger ITP and many pro-inflammatory mediators are involved in ITP [2,32]. In this regard, platelet-pathogen interactions and pro-inflammatory platelet functions could be of potential interest for further ITP studies. Binding of bacterial lipopolysaccharide (LPS) to platelet TLR4, for example, has been shown to induce thrombocytopenia in mice in the presence of anti-platelet antibodies [33,34]. This mechanism could probably be involved in the exacerbations of thrombocytopenia that are observed in patients with ITP during or after infections. The immune functions of platelets may also be involved in promoting inflammation in ITP. As such, platelets in ITP have been suggested to be more vulnerable to oxidative stress because of ITP-related reduced intracellular antioxidant capacity [35]. Due to this impaired function, ITP platelets showed a higher expression and activation of the NLRP3 inflammasome [35]. Subsequently, this leads to increased pyroptosis, a newly recognized form of programmed cell death, in platelets [35]. Furthermore, the binding of C-reactive protein (CRP), an acute phase protein upregulated in acute infections and inflammation, to platelets drives another pathway leading to thrombocytopenia in ITP. Increased levels of CRP are observed in ITP [36]. Binding of autoantibodies to platelets was shown to lead to oxidative changes resulting in phosphorylcholine exposure from the platelet- membrane, providing a calcium-dependent binding site for CRP which then enhanced antibody-mediated platelet phagocytosis via FcγRs [36]. CRP- and autoantibody-opsonized platelets are thus more potently phagocytized via phagocytic FcγRs leading to low platelet levels in the circulation [36].

### 2.3. Platelets and (Auto)-Immune Responses in ITP

Platelets can directly interact with both lymphoid and myeloid cells through various platelet-receptors (e.g., CD40L, P-selectin, TLR, MHC class I) and through several platelet-derived chemo- and cytokines [12,14,32]. We propose that these platelet-immune cell interactions might contribute to the autoimmune response in ITP. CD40L, for example, is a costimulatory protein that can enhance CD8+ T cell responses and activate several B cell responses such as Ig class switching and memory B cell formation [17,32]. Platelets express both membrane-bound CD40L and secrete a soluble form of CD40L (sCD40L) [17]. Platelets are the main source of sCD40L, as they produce over 90% of the sCD40L in the circulation [17]. Platelet-associated expression of CD40L is increased in ITP and elevated levels of sCD40L are found in approximately 60% of patients with ITP [32,37,38]. Interestingly, platelet-CD40L has been shown to activate B cells from patients with ITP in vitro resulting in the production of GPIIb/IIIa antibodies [37]. These results suggest that platelets are able to participate in the autoreactive immune response in ITP through expression of CD40L. Some early trials with anti-CD40L monoclonal antibodies have demonstrated some efficacy in patients with refractory ITP [39]. Although the development of the molecules used in these trials has been discontinued due to safety concerns, newer anti-CD40L antibodies are currently under investigation for ITP [40,41]. Autoantigen presentation by platelet MHC class I receptors might be another pathway contributing to autoimmunity in ITP. Murine ITP models revealed that megakaryocytes express self-antigens through their MHC-class I receptor which led to CD8+ T cell mediated thrombocytopenia [42]. Platelets themselves have also been shown to activate antigen-specific CD8+ T cells during sepsis through presentation of exogenous peptides via their MHC class I receptors [43,44]. Further research should determine whether platelets in ITP express these self-peptides as well. Besides sCD40L, other platelet-derived mediators such as platelet factor 4 or serotonin might also affect the immune response in ITP [45,46]. 

## 3. Hypothesis 2: Platelet Microparticles in ITP

**Figure 2:**
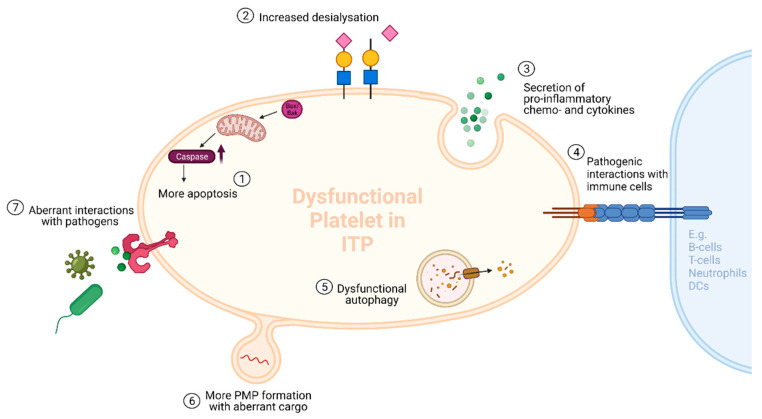
Platelet functions that may be altered or dysfunctional in ITP. (**1**) Platelet apoptotic activity is known to be increased in ITP [56] (**2**) Increased desialylation of platelets has been reported in refractory patients with ITP leading to enhanced Fc-independent platelet clearance in the liver [3,10,20]. (**3**) Platelets may secrete pro-inflammatory mediators such as chemokines and cytokines into the circulation in ITP (see main text). (**4**) Platelets may modulate the immune response in ITP through interaction with various immune cells such as B cells, T cells, neutrophils and dendritic cells (DCs) (see main text). (**5**) Platelet-autophagy has been suggested to be dysfunctional in ITP [57] (**6**) Platelets in ITP might release more PMPs and PMPs with aberrant cargo (see main text). (**7**) Platelets may interact with pathogens in an aberrant way in ITP leading to more inflammation and exacerbation of thrombocytopenia, e.g., through aberrant stimulation of platelet Toll-like receptors (see main text). Created with BioRender.com.

Platelets are able to influence immune responses systemically by shedding platelet microparticles (PMPs) and releasing them into the circulation [12,14,15]. PMPs are extracellular vesicles of 0.1–1.0 μm that are shed by platelets during platelet activation, apoptosis or exposure to (shear) stress [47,48]. PMPs are found to be the most abundant type of microparticles, representing 70–90% of all cell-derived microparticles in the circulation [49]. Membrane-bound PMPs can carry surface markers (e.g., P-selectin, CD41) from their parent cell and contain intracellular cargo (such as RNA and mitochondrial DNA) [12,14,15]. In this way, PMPs can interact with many different cell types and influence their cellular responses [12,14,15]. ITP has been associated with higher PMP levels [50,51] and previous reports have primarily focused on their hemostatic role, proposing that their elevated levels in ITP may be a compensatory mechanism to reduce bleeding [50]. Interestingly, however, accumulating literature suggests that PMPs also have immune-modulating functions and might be involved in the development of autoimmune mediated diseases such as SLE and RA [52,53]. Furthermore, PMPs were shown to infiltrate the bone marrow during inflammation and to alter megakaryopoiesis [54,55]. Given these findings, we hypothesize that PMPs play a role in the pathogenesis of ITP in two ways: (1) by stimulating immune cells such as B cells, T cells and monocytes in a pro-inflammatory manner and (2) by impairing the function of megakaryocytes in the bone marrow which causes defects in the platelet production (see Figure 1 and Figure 2). 

### 3.1. The First Hit

An initial trigger is required for platelets to shed PMPs—a first hit. Platelets have been observed to shed microparticles from their membrane during platelet activation and platelet apoptosis [47,48]. In ITP, a (viral) infection often precedes the onset of the disease, especially in children [58,59]. Such a preceding infection could be an initial trigger for platelets to release PMPs. In sepsis, bacterial LPS can also activate platelets via TLR-4 to shed PMPs [60]. Furthermore, bacterial components and epitopes of influenza virus can form immune complexes in immunized individuals [14]. Binding of these immune complexes to the platelet IgG Fc receptor FcγRIIa stimulates the platelet to form PMPs [14]. Of interest, this platelet FcγRIIa-mediated response may also be paralleled by the secretion of serotonin [61,62]. Serotonin can drive naïve T cells to activate and proliferate and has been suggested to orchestrate neutrophil responses in sepsis [17,25,61]. We regard the recognition of PAMPs and DAMPs by platelet-TLR and the binding of immune complexes to FcγRIIa as two likely pathways for the initial release of PMPs in ITP. Nonetheless, most patients with chronic ITP have no documented history of recent infections and in self-limiting ITP, a preceding pathogen is usually not found [63]. Therefore, other triggers for PMP release should also be considered. One could hypothesize that PMP release in ITP is a consequence of earlier immunological changes. Binding of anti-GPIbα and anti-GPIIb/IIIa, the two most commonly detectable platelet antibodies in ITP, can cause platelet activation which can lead to enhanced Fc-dependent platelet clearance [10,64]. Hence, autoantibodies could be a likely trigger for platelets to shed PMPs in ITP. In this regard, injection with immune complexes has been shown to trigger PMP release in mice expressing the FcγRIIA transgene [65]. Other triggers have been described to activate platelets, such as shear forces from the blood flow and activation of glycoprotein VI [14,66]. Glycoprotein VI is a major receptor for collagen and has been shown to trigger the formation of PMPs in patients with RA [15]. In addition, some cases of ITP with anti-GPVI antibodies have been reported [67,68]. Whether the aforementioned mechanisms cause platelets to generate PMPs in ITP should be further evaluated and genetic alterations, e.g., for the FcγRIIa, should be taken into consideration

### 3.2. ITP-PMPs: How and Why Are They so Different?

Through the first hit, platelets are triggered to generate PMPs. We postulate that PMPs in ITP differ from healthy PMPs in two ways: (1) the number of PMPs in ITP is increased and (2) ITP-PMPs carry aberrant cargo.

#### 3.2.1. Increased Levels of PMPs in ITP

PMP levels are found to be increased in patients with ITP [50,51]. These findings are consistent with other autoimmune mediated disorders, as higher levels of PMPs have also been observed in SLE and RA [52,69,70]. Interestingly, PMPs are suggested to be rapidly cleared from the circulation through mechanisms that are not yet understood, but may involve lactadherin [71,72]. Increased levels of PMPs were found in lactadherin deficient mice and their splenic macrophages demonstrated a decreased phagocytic capacity [73]. After platelet transfusion in patients with severe thrombocytopenia undergoing hematopoietic stem cell transplantation, a half-life of 5.3–5.8 h for PMPs was observed [71]. Thus, if PMP production is initially infection-related, other mechanisms could be responsible for the continuous elevated PMP levels in patients with chronic ITP after clearance of the pathogen. Autoantibodies or other immunological changes could induce platelet activation and possibly more PMP shedding. Furthermore, we hypothesize that the immune functions of platelets are altered in ITP resulting in sustained elevated levels of PMPs due to continued triggering and/or defective clearance of PMPs (see Section 3.4). Whether the increased PMP release in ITP is driven by autoimmune induced changes and/or by alterations in the immune functions of platelets is, however, a topic of further investigation.

In addition to PMPs, megakaryocytes are also known to release microparticles (MK-MPs) [73,74]. Since platelets and megakaryocytes share several common markers (e.g., CD41), a distinction between platelet- and megakaryocyte-derived MPs has not always been made. Some studies claim that the majority of microparticles in the circulation are megakaryocyte-derived instead of platelet-derived, while others hypothesize that both sources contribute to the total amount equally [73,74]. Animal models have revealed that MK-MPs are formed concomitantly with pro-platelet production [75]. In ITP, platelet production and megakaryocyte maturation may be impaired while the number of megakaryocytes appears to be increased or normal [76,77]. Possibly, the higher number of megakaryocytes could contribute to the higher levels of circulating MPs, although more research is needed to confirm during which step of megakaryopoiesis MPs are released. Autoantibodies targeting the MKs in the bone marrow could be another possible mechanism for MK-MP formation in ITP, since autoantibodies may interfere with normal proplatelet formation and platelet release [78]. Recently, murine ITP models revealed that CD8+ T cells may also affect megakaryocyte function [79]. However, studies regarding MK-MP formation in ITP are lacking and are warranted to assess their contribution to the total levels of MPs.

#### 3.2.2. The Different Cargos of ITP-PMPs

PMPs are thought to mediate cell-to-cell communication by carrying a large variety of substances such as nucleic acids, cytokines and chemokines, functional enzymes, surface receptors, transcription factors, autoantigens and mitochondria [12,14]. We hypothesize that the numbers of PMPs are not only increased in ITP, but that the PMP cargo may be altered as well. Emerging lines of evidence have demonstrated that several important PMP components such as microRNA (miRNA) are aberrant in ITP [80,81]. In addition, platelets in ITP are known to have several dysfunctional properties such as increased apoptotic activity, autophagy deficits and increased desialylation (Figure 2) [20,56,57]. Hence, it is plausible that platelets in ITP have aberrant intra- and extracellular compounds that they can transfer to their PMPs. Furthermore, PMPs have been reported to enhance megakaryopoiesis in thrombocytopenic mice with acute liver injury [55]. Despite the increased number of PMPs in ITP, megakaryopoiesis appears to be impaired, implying that the PMP cargo could be altered in ITP. 

Given their key role in gene expression regulation, miRNAs are amongst the likely PMP cargo-candidates to be involved in ITP. Although platelets are anucleated cells, they contain significant amounts of RNA which they are able to transfer into their microparticles [18,82]. MiRNAs are small non-coding RNA molecules that regulate gene expression by binding to target-mRNAs and inducing translation inhibition [82]. Aberrant miRNA expression has been associated with ITP, although their exact pathogenic role remains unclear [83,84]. Interestingly, several miRNAs with significant immunological functions have been described to be up- or down regulated in ITP [81,84,85,86]. Moreover, circulating miRNA levels have been observed to be increased or decreased after several ITP treatments, indicating that miRNA could be a possible biomarker for therapeutic response [30,87,88]. In this regard, one could hypothesize that PMP contents such as miRNAs might even differ between several subgroups of patients with ITP, including acute vs. chronic ITP and therapeutic responders vs. non-responders. These differences could possibly contribute to the heterogeneity between patients with ITP. One important shortcoming of the aforementioned miRNA studies is, however, that miRNAs are mostly measured as freely circulating in plasma and not specifically measured inside PMPs. Recently, however, a study by Sun et al. identified the miRNA profile of plasma-derived microparticles in ITP and found them to be different from those of healthy controls [80]. Since the clinical value of the aforementioned findings is not yet fully understood, future research should be focused on the types of miRNAs found in ITP-PMPs and the specific roles they may fulfil. Aside from miRNAs, PMPs are able to carry several other significant cargo which could impact target cell functions in ITP. Mitochondrial DNA (mtDNA), for instance, has been described to be engaged in inflammatory cytokine production and autoimmunity, particularly in the presence of autophagy deficits [89]. Additionally, several platelet-derived chemokines, cytokines and mediators are carried by PMPs and have been described to be involved in diverse cellular interactions and responses [15,90].

In summary, we hypothesize that ITP-PMPs might carry aberrant molecules, such as different types of miRNA, compared with healthy PMPs. Furthermore, the content of PMPs may differ between ITP patient groups and could contribute to heterogeneity in ITP.

### 3.3. Hypothesis 2a: The PMP-Immune Cell Interaction Hypothesis

Unlike platelets, PMPs are readily able to enter the lymphatic system and the bone marrow, where they can modulate target cells [65]. PMPs have been described to exert a pro-inflammatory function in autoimmune diseases [69,91]. In patients with RA, PMPs were demonstrated to directly contribute to joint inflammation and disease activity [91]. Together with the increased levels of PMPs seen in ITP, these observations suggest that PMPs might also participate in the altered immune response in ITP. Studies stratifying PMP levels for subgroups of patients with ITP, e.g., acute vs. chronic ITP or therapeutic responders vs. non-responders, have been scarce. Tantaway et al. found that within their pediatric cohort (*n* = 40), patients with acute ITP, had the highest levels of PMPs compared with patients with chronic ITP or healthy controls [50]. These results support the hypothesis that higher PMP levels in newly diagnosed ITP may correlate with more immune dysregulation and higher disease activity. However, two other cohort studies in adult patients with ITP (*n* = 29 and *n* = 76) found no difference in PMP levels between splenectomized and non-splenectomized patients. Moreover, no correlation between platelet counts and PMP levels were found [51,92]. Although one could expect PMP levels to decrease during remission, this phenomenon may be explained by the fact that splenectomy does not tackle the cause of ITP (loss of immune tolerance) but rather takes away the major destruction site of platelets. Therefore, splenectomized patients with ITP are likely to still have high levels of PMPs, while their platelet counts may increase due to less platelet destruction. More research into PMP levels and contents for specific ITP subgroups is needed to shed light on this topic.

Our hypothesis that PMPs might contribute to the altered immune response in ITP is further endorsed by observations of interactions between PMPs and both lymphoid and myeloid cells. PMPs were shown to induce NET formation in patients with acute pancreatitis [93]. In SLE, PMPs can bind autoantibodies to form immune complexes, stimulating monocytes to exhibit pro-inflammatory responses [69]. In addition, PMPs have been shown to induce antigen-specific IgG production by transferring CD40L to B cells in mice after immunization with adenovirus [94,95]. Although this has not been investigated in ITP, it would be interesting to explore whether ITP-PMPs can induce autoantibody production or monocyte activation and if ITP-PMPs are also able to form immune complexes by binding to autoantibodies. PMPs have also been reported to impair Treg differentiation [96]. As Tregs can be regarded as key players in the loss of immune tolerance in ITP, this could be an interesting pathway to explore in future studies. Vesicles from bone marrow mesenchymal stem cells have already been shown to regulate the imbalance of Th17/Tregs in ITP through the delivery of miRNA-146a-5p [97]. One could hypothesize that a comparable pathway might exist for PMPs in ITP since PMPs are known to carry miRNA. However, substantially more evidence is needed to support this theory, which could be gathered from larger cohort studies investigating the effects of PMPs on different T cell subsets. A recent study from Marcoux et al. showed that PMPs, similar to platelets themselves, present antigens via their MHC class I receptor to CD8+ T cells [65]. They suggest that platelet-antigens may also be presented by PMPs, which might contribute to the T cell mediated anti-platelet response in ITP as well [65]. In summary, PMPs may contribute to the altered immune balance and the loss of immune tolerance in ITP through interactions with myeloid and lymphoid cells. In addition, the higher levels of PMPs in ITP might correlate with a higher disease activity, although more research is needed to establish this association in ITP. 

### 3.4. Hypothesis 2b: The PMP-Bone Marrow Hypothesis

PMPs can infiltrate the bone marrow during inflammatory conditions and interact with and reprogram megakaryocytes [54,55,98]. In mice with acute liver injury-induced thrombocytopenia, PMPs have been demonstrated to promote megakaryopoiesis leading to elevated platelet production [55]. In addition, it was shown that PMPs transfer specific miRNAs (e.g., miRNA-1915–3p) to MKs leading to MK proliferation, differentiation and platelet production [11,55]. As PMPs of patients with ITP are thought to carry an aberrant profile of miRNAs compared with healthy individuals, we postulate that PMPs in ITP may affect MKs differently. Exosomes are extracellular vesicles (30–150 nm) which are considerably smaller than MPs (100–1000 nm) are derived from the endosomal compartment of cells instead of the cellular membrane [94,99]. Exosomes obtained from ITP plasma have been shown to inhibit apoptosis of MKs by activating the Bcl-xl/caspase pathway in vitro [98]. Since MK apoptosis is essential for platelet production, platelet release was significantly reduced in these samples. These findings are supported by earlier studies showing a decreased population of apoptotic MKs in patients with chronic ITP [76,100]. Besides suppressing MK apoptosis, ITP plasma has also been reported to induce MK autophagy [101]. Autophagy is a complex multi-step intracellular process aiming to eliminate and recycle cellular components [102]. In patients with ITP, abnormal autophagy inhibits MK differentiation and leads to the production of larger but fewer platelets, severely impairing platelet production [102]. Which mechanisms cause the promotion of MK autophagy after exposure to ITP plasma is still unclear. However, since miRNAs are known to be involved in the regulation of autophagy, involvement of PMPs in the MK autophagy abnormalities seen in ITP could be possible [103]. Altogether, we hypothesize that PMPs may affect MKs in the bone marrow leading to deficits in platelet production by megakaryocytes in ITP. Further research should focus which and how MK functions may be altered by PMPs in ITP. Additionally, we propose that PMPs alter bone marrow-megakaryopoiesis in ITP in such way that dysfunctional platelets are produced (Figure 1). These dysfunctional platelets might be more easily cleared from the circulation due to their altered platelet functions (Figure 2). Moreover, we postulate that the shedding of PMPs is amongst the platelet functions that could be altered in ITP. More PMPs and PMPs with altered cargo are produced by these ITP dysfunctional platelets leading to a positive feedback loop (Figure 1). In this way, PMPs might not only contribute to the disrupted immune response in ITP, but might also promote their own production by altering platelet production by MKs.

## 4. Final Recommendations

Platelets are able to promote inflammation and to drive immunological responses either directly through their receptors or indirectly by releasing platelet-derived molecules such as chemokines and PMPs. These immune modulating properties may likely be involved in various aspects of ITP pathogenesis. Several hypothetical pathways about the immunological roles of platelets in ITP are suggested in this review and it would be interesting to address them in further studies (Table 1). As the mechanisms mentioned in this review are mainly based on murine studies, one should be cautious when translating these murine models to human research. Although animal models of ITP can be clinically relevant and are an excellent means to further dissecting the pathophysiologic mechanisms underlying ITP, the differences between mice and human physiology, e.g., wild-type conventional mice lacking platelet-FcγRIIa, should be taken into account [104]. Future research should focus on platelet-receptors and platelet-derived molecules and their interactions with inflammatory mediators and immune cells in ITP. Furthermore, large cohort studies comparing the differences in PMP levels and contents between patients with acute ITP, chronic ITP or patients in remission with or without therapy are needed to clarify the immune modulating role of PMPs in ITP. In addition, the in vitro and in vivo effects of PMPs on megakaryocytes in ITP and immune cells such as T cells, B cells and monocytes should be further explored. Most importantly, the likely possibility that platelets are not only the victims of the autoimmune response in ITP, but also immuno-modulating cells involved in promoting and sustaining their own destruction, is an important issue which warrants further investigation. More understanding of the role of platelets in the pathophysiology of ITP could help us with identification of new diagnostic biomarkers and predictors of therapeutic response [87]. Ultimately, this may lead to new platelet-directed therapeutic strategies for ITP.

## Figures and Tables

**Table 1:** The key hypotheses of this review.

Key Hypotheses in the Pathogenesis of ITP
**Hypothesis 1: Platelets and their immune functions in ITP**
1. Platelets promote inflammation and drive pathogenic immuno-modulatory responses.
**Hypothesis 2: PMPs in ITP**
2a. Platelets shed PMPs which interact with immune cells and stimulate pathogenic immuno-modulatory responses
2b. Platelets shed PMPs which impair bone marrow megakaryopoiesis and cause deficits in platelet number and function.
